# Role of glucagon-like peptide-1 receptor agonists in Alzheimer’s disease and Parkinson’s disease

**DOI:** 10.1186/s12929-024-01090-x

**Published:** 2024-11-05

**Authors:** Chien-Tai Hong, Jia-Hung Chen, Chaur-Jong Hu

**Affiliations:** 1 Department of Neurology, School of Medicine, College of Medicine, Taipei Medical University, No. 250, Wuxing St., Xinyi Dist., Taipei, 110 Taiwan; 2 Department of Neurology, Shuang Ho Hospital, Taipei Medical University, New Taipei City, Taiwan; 3 Taipei Neuroscience Institute, Taipei Medical University, Taipei, Taiwan

**Keywords:** Neurodegenerative diseases, Alzheimer’s disease, Cognition, Parkinson’s disease, Diabetes, Glucagon-like peptide-1 receptor agonists, Neuroprotection

## Abstract

Neurodegenerative diseases, including Alzheimer’s Disease (AD) and Parkinson’s Disease (PD) are common complications of diabetes, arising from insulin resistance, inflammation, and other pathological processes in the central nervous system. The potential of numerous antidiabetic agents to modify neurodegenerative disease progression, both preclinically and clinically, has been assessed. These agents may provide additional therapeutic benefits beyond glycemic control. Introduced in the twenty-first century, glucagon-like peptide-1 receptor agonists (GLP-1RAs) are a class of antidiabetic drugs noted not only for their potent glucose-lowering effects but also for their cardiovascular and renal protective benefits. Various GLP-1RAs have been demonstrated to have significant benefits in in vitro and in vivo models of neurodegenerative diseases through modulating a variety of pathogenic mechanisms, including neuroinflammation, autophagy, mitochondrial dysfunction, and the abnormal phosphorylation of pathognomonic proteins. These agents also have substantial protective effects on cognitive and behavioral functions, such as motor function. However, clinical trials investigating GLP-1RAs in diseases such as AD, PD, mild cognitive impairment, psychiatric disorders, and diabetes have yielded mixed results for cognitive and motor function. This review examines the link between diabetes and neurodegenerative diseases, explores the effects of antidiabetic agents on neurodegeneration, provides a concise overview of the GLP-1 pathway, and discusses both preclinical and clinical trial outcomes of GLP-1RAs for neurodegenerative diseases, including their effects on cognition in AD and PD. This review also proposed new strategies for the design of future clinical trials on GLP-1 RAs for both AD and PD.

## Introduction

### Alzheimer’s disease (AD)

The global increase in the prevalence of neurodegenerative diseases, particularly AD and PD, as a result of an aging population poses a significant challenge to health-care systems worldwide [1, 2]. AD is the most common cause of dementia which is characterized by progressive cognitive decline and neurodegeneration. The hallmark pathologies in AD are the accumulation of amyloid-beta (Aβ) plaques and tau tangles in the brain, leading to neuronal loss and brain atrophy [3, 4]. The risk of AD significantly increases with age, particularly after 65 years old [5]. Given the global trend of population aging, the number of individuals living with AD is expected to increase remarkedly, necessitating a shift in the care provided to these individuals and the resources re-allocated [6].

### Parkinson’s disease (PD)

PD is another prevalent neurodegenerative disorder; it primarily affects motor function due to the loss of dopamine-producing neurons in the substantia nigra, a region of the midbrain. Its symptoms include tremors, rigidity, bradykinesia (slowness of movement), and postural instability. Similar to AD, the prevalence of PD increases with age and is expected to increase with population aging [7]. Cellular senescence, oxidative stress, and mitochondrial dysfunction are common aging-related processes that exacerbate the neurodegenerative pathways in both diseases [8, 9]. The increased prevalence indicates the need for novel therapeutic strategies and supportive care to manage the symptoms of these neurodegenerative diseases and to improve the quality of life for the affected individuals.

### Urgent need of treatment for AD and PD

Despite considerable advances, existing AD and PD treatments primarily address the symptoms rather than the cause. Disease-modifying therapies that can halt or slow the progression of these neurodegenerative disorders are urgently required. Researchers are currently focusing on the molecular and cellular mechanisms underlying AD and PD, which are key to targeted therapies. Therapies for AD should reduce Aβ accumulation, prevent tau pathology, and protect neuronal function. For PD, the aim is to protect dopaminergic neurons, improve mitochondrial function, reduce α-synuclein accumulation and formulate gene therapies. However, these diseases are difficult to treat given their complexity and the challenges of drug delivery to the brain [10, 11].

## Diabetes and neurodegenerative diseases

A growing body of evidence supports the link between diabetes and an increased risk of neurodegenerative diseases such as AD and PD. This link is believed to be primarily mediated by the metabolic, vascular, and inflammatory pathways associated with diabetes, which contribute to the pathogenesis of neurodegeneration. The pathways include inflammation, vascular damage, mitochondrial dysfunction, the effects of insulin resistance (IR) on metabolism in the brain, the formation of advanced glycation end products (AGEs), and the aggregation of pathological proteins such as Aβ and α-synuclein [12].

### Diabetes and AD

Some researchers have termed AD “type 3 diabetes,” reflecting the observed link between IR (a hallmark of type 2 diabetes) and AD [13]. IR in the brain impairs glucose metabolism, which is critical for the provision of neuronal energy and the maintenance of synaptic function. This impairment can lead to neuronal damage and increased Aβ accumulation, a key feature of AD pathology [14, 15]. In diabetes, high glucose levels lead to the formation of AGEs, which can accumulate in the brain and contribute to Aβ aggregation and tau phosphorylation, both of which play pivotal roles in AD pathology. AGEs promote oxidative stress and inflammation, leading to the further damage of neurons [16]. Diabetes causes vascular damage through atherosclerosis, leading to impaired blood flow and reduced oxygen supply to the brain. This vascular dysfunction may cause the development of cerebral amyloid angiopathy and may contribute to the neurodegenerative processes observed in AD [17].

### Diabetes and PD

Diabetes induces mitochondrial dysfunction, which is a key feature of PD pathology [18, 19]. Impaired mitochondrial function in dopaminergic neurons in the substantia nigra can lead to increased oxidative stress, neuronal damage, and neuronal death, contributing to PD progression [20]. Chronic systemic inflammation, a consequence of diabetes, can exacerbate neuroinflammation, which is implicated in the pathogenesis of PD. Inflammatory mediators can cross the blood–brain barrier, activating the microglia and leading to the release of neurotoxic factors with adverse effects on dopaminergic neurons [21]. Evidence suggests that diabetes exacerbates the aggregation of α-synuclein, a protein that accumulates in the brain in patients with PD, forming Lewy bodies. Hyperglycemia and IR have been linked to increased α-synuclein aggregation, further implicating the role of diabetes in the pathogenesis of PD [22].

### Diabetes and pathogeneses of AD and PD

The evidence linking diabetes to the increased risks of AD and PD underscores the complex mechanisms through which metabolic diseases influence brain health and function. Understanding these links is crucial for developing preventive strategies and treatments that address the metabolic basis of neurodegeneration. Moreover, the aforementioned findings highlight the importance of managing diabetes not only to prevent its physical health complications but also to mitigate the risk of neurodegenerative diseases.

**Fig. 1:**
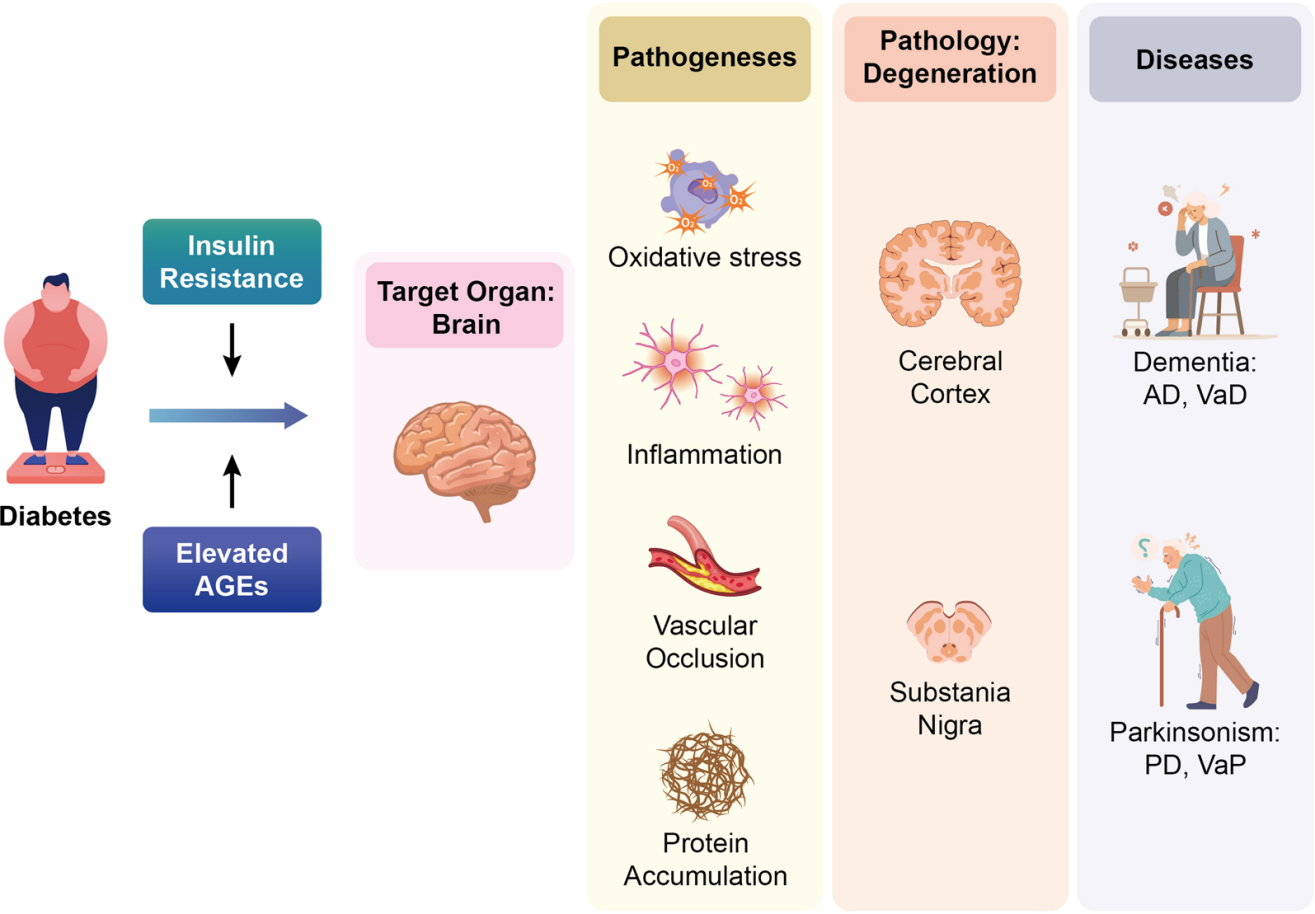
The postulated mechanism by which diabetes contributes to neurodegeneration in Alzheimer’s disease (AD) and Parkinson’s disease (PD) may involve the accumulation of advanced glycation end products (AGE) and cellular insulin resistance (IR). For the target organ, brain, these two phenomenon lead to inflammation, vascular occlusion, oxidative stress, and the aggregation of pathognomonic proteins, which, in turn, trigger neurodegeneration in the cerebral cortex for dementia, such as AD and vascular dementia (VaD), and the substantia nigra for parkinsonism, such as PD and vascular parkinsonism (VaP)

In summary, the potential mechanism by which diabetes contributes to neurodegeneration in AD and PD may involve the accumulation of AGE and cellular IR. This leads to neuroinflammation, vascular occlusion, mitochondrial dysfunction, and the aggregation of pathognomonic proteins, which, in turn, trigger neurodegeneration in the cerebral cortex for AD and the substantia nigra for PD (illustrated in Fig. 1).

## Neuroprotective effect of antidiabetic agents

### Metformin and neuroprotection

Considering the link between diabetes and the increased risks of AD and PD, researchers have investigated the possible neuroprotective effect of antidiabetic agents. Epidemiological evidence has increasingly suggested that metformin, a commonly prescribed medication for type 2 diabetes, protects against the development of neurodegenerative diseases [23]. One candidate mechanism underlying this neuroprotective effect lies in the ability of metformin to improve insulin sensitivity, reduce chronic inflammation, and exert direct effects on brain cells [24]. Furthermore, research has indicated that metformin may improve cognitive performance in individuals with type 2 diabetes [25]. This cognitive benefit may stem from metformin’s ability to improve glucose metabolism in the brain and reduce IR, which are the risk factors for AD. Epidemiological studies have also demonstrated that the use of metformin in individuals with type 2 diabetes is associated with a lower risk of PD [26]. This association suggests that the metabolic benefits of metformin extend to the protection of dopaminergic neurons. Compelling epidemiological evidence links metformin prescription to the reduced risks of AD and PD, indicating the potential repurposing of this widely used diabetes drug for neuroprotection. A clinical trial investigated the effect of 8-week metformin treatment on aging individuals with mild cognitive impairment (MCI) or early AD, and the results revealed that metformin is associated with improved executive function, companied by improvement in learning/memory and attention [27]. However, more large-scale randomized controlled trials (RCTs) are required to verify the neuroprotective effects of metformin.

### Thiazolidinediones (TZDs) and neuroprotection

TZDs, including pioglitazone and rosiglitazone (which are often referred to by the broader term “thioglitazones” [TZDs]), are a class of medications primarily used to treat type 2 diabetes. They function as insulin sensitizers by activating the peroxisome proliferator-activated receptor gamma (PPAR-γ), a type of nuclear receptor that plays a crucial role in the regulation of glucose and lipid metabolism. Epidemiological research has been conducted to examine the potential link between the prescription of TZDs and the reduced risks of AD [28, 29] and PD [30, 31], and studies have explored their anti-inflammatory and neuroprotective effects [32]. A few RCTs have investigated the effects of pioglitazone on cognitive decline in patients with AD [33–36]. A pilot study demonstrated that pioglitazone led to cognitive and metabolic improvements in patients with diabetes with AD or MCI [34]. Nevertheless, a phase III study found no significant differences in cognition between rosiglitazone treatment and placebo in APOE-ε4-non-carriers or overall [37]. Early trials have provided implication of the neuroprotective benefits of pioglitazone, which are potentially mediated by its anti-inflammatory and insulin-sensitizing effects. Varied results have been obtained, and more robust and larger-scale RCTs are necessary to draw definitive conclusions. Some clinical trials have been conducted on the repurposing of pioglitazone for PD treatment. A notable RCT is the pioglitazone in early PD trial, which failed to demonstrate the possibility of pioglitazone delaying disease progression in early-stage PD patients [38]. The repurposing of metformin or TZDs for neuroprotection in AD and PD remains a promising, but not yet conclusively proven, strategy. Although both drug classes could theoretically offer neuroprotective benefits based on their mechanisms of action, RCTs have provided mixed or inconclusive results. Thus, more targeted, well-designed clinical trials are required for providing clearer evidence on the efficacy, optimal dose, and safety profiles of these drugs in AD and PD. Table 1 summarized the clinical trials of metformin and TZDs on cognition, MCI, AD, and PD.

**Table 1:** Summary of published clinical trials of metformin and thiazolidinedione on cognition in mild cognitive impairment (MCI), Alzheimer’s disease (AD), and Parkinson’s disease (PD)

Anti-diabetic agents [Ref.]	Population	Treatment duration	Phase	Outcome measurements (primary**/**secondary)	Final results
Metformin [27]	Age 55–80, MCI or early AD (CDR ≤ 1.0), MMSE > 19, at least one positive AD biomarker	8 weeks	2	CSF Aβ, tau, p-tau/ADAS-Cog, CANTAB, TMT-B, DS, BNT, ASL-MRI	No change of CSF biomarkers, improvement executive function
Pioglitazone [34]	Mild to moderate AD (MMSE 16–26) and amnestic MCI with DM	6 months	NA	MMSE, ADAS-JCog, WMS-R, FAB, CF, rCBF	Improvement of ADAS-Jcog scores
Rosiglitazone [37]	Age 50–90, probable AD (MMSE 10–23)	24 weeks	3	ADAS-Cog, CIBIC+/NPI, DAD, MMSE	No evidence of efficacy
Pioglitazone [36]	Probable AD, MMSE 12–26, CDR 1 or 2, with cholinesterase inhibitors	6 months	NA	CDR-SB, ADAS-Cog, NPI	No significant treatment effect
Pioglitazone [33]	Age 65–83, CDR 0, MMSE ≥ 25, CVLT-LDFR or BVMT-DR > − 1.5 SDs	5 years	3	Time to diagnosis of MCI/ADCS-ADL	No delay the onset of MCI
Pioglitazone [38]	Age > 30, PD < 5 years, H&Y ≤ 2	44 weeks	2	UPDRS/PDQ-39, SEADL, UPSIT	No significant treatment effect

*ADAS-Cog* Alzheimer’s disease assessment scale-cognitive subscale, *ADAS-JCog* Alzheimer disease assessment scale (Japanese version) cognitive subscale, *ADCS* Alzheimer’s disease cooperative study, *ADL* activities of daily living, *ASL* arterial spin labeling, *BNT* Boston naming test, *BVMT-DR* brief visuospatial memory test-revised, *CANTAB* Cambridge neuropsychological test automated battery, *CDR* clinical dementia rating, *CDR-SB* clinical dementia rating-sum of boxes, *CF* category fluency, *CIBIC+* clinician’s interview-based impression of change with caregiver’s input, *CSF* cerebrospinal fluid, *CVLT-LDFR* California verbal learning test-long delay free recall, *DAD* disability assessment for dementia, *DS* digit span, *DM* diabetes mellitus, *FAB* frontal assessment battery, *H&Y* Hoehn and Yahr stages, *MCI* mild cognitive impairment, *MRI* magnetic resonance imaging, *NPI* neuropsychiatric inventory, *PDQ-39* Parkinson’s disease questionnaire-39, *rCBF* regional cerebral blood flow, *SEADL* self-efficacy for activities of daily living, *TMT* trail making test, *UPDRS* unified Parkinson’s disease rating scale, *UPSIT* University of Pennsylvania smell identification test, *WMS-R* Wechsler memory scale-revised

## Overview of glucagon-like peptide-1 and its role in glucose metabolism

New-generation antidiabetic agents, particularly those targeting the glucagon-like peptide-1 (GLP-1) pathway, represent a significant advancement in the treatment of type 2 diabetes. GLP-1 is an incretin hormone that is secreted in response to food intake, and it exerts multiple actions conducive to glycemic control, including enhancing glucose-dependent insulin secretion, suppressing glucagon release, and delaying gastric emptying [39]. GLP-1 receptor agonists (GLP-1RAs) are designed to mimic the action of endogenous GLP-1 but have a longer duration of action, making them effective therapeutic agents for type 2 diabetes [40]. Studies have demonstrated the efficacy of GLP-1RAs for glycemic control and their potential cardiovascular [41] and renal protective effects [42]. These effects are particularly relevant given the increased risks of cardiovascular and kidney diseases in patients with diabetes. Several landmark cardiovascular outcome trials have demonstrated that GLP-1RAs can significantly reduce the risk of major adverse cardiovascular events (MACE), which include cardiovascular death, nonfatal myocardial infarction (heart attack), and nonfatal stroke [43]. The cardiovascular benefits of GLP-1RAs extend beyond their effects on blood glucose levels. Potential mechanisms include improvements in blood pressure, lipid profiles, body weight, inflammation, and endothelial function as well as direct cardioprotective effects on the cardiac muscle and vasculature [41]. GLP-1RAs can reduce the incidence of MACEs and delay the progression of kidney disease; thus, they are an attractive therapeutic option for patients with type 2 diabetes, especially those with existing cardiovascular disease or at high risk for those complications.

## Molecular mechanisms of GLP-1 agonists for neuroprotection

GLP-1 receptors are functionally expressed in insulin-releasing neuroglia form cells in the rat cerebral cortex, and hyperglycemia significantly increases GLP-1 receptor expression. Additionally, GLP-1 receptor activation by GLP-1RAs can mimic effects on these cells similar to those on pancreatic beta cells, suggesting that this pathway also modulates neural insulin production [44].

### Insulin and GLP-1 signaling pathway

The molecular mechanism of neuroprotection of several GLP-1RAs has been explored using in vivo and in vitro models. Existing findings indicate that the protective mechanism is mainly attributed to the GLP-1 signaling pathway, which involves cyclic adenosine monophosphate (cAMP), protein kinase A (PKA) and cAMP response element-binding protein (CREB). A study demonstrated the neuroprotective effects of liraglutide, a commercially available GLP-1RA, on neuronal cells from H_2_O_2_-induced oxidative stress and glutamate-induced excitotoxicity, enhancing cell viability. The neuroprotective effects are linked to the activation of the cAMP/PKA/pCREB pathway [45]. The neuroprotective benefits of GLP-1RAs may also result from the insulin signaling pathway. Exendin-4 (Ex-4), another GLP-1RA that is homologous to commercially available exenatide, ameliorated IR in primary neurons through insulin receptor substrate-1 (IRS-1), AKT, and glycogen synthase kinase-3β (GSK-3β) pathways; GSK-3β significantly contributes to neuroprotection given its role in tau hyperphosphorylation in AD pathology [46].

### Mitochondria and anti-oxidants

The neuroprotection of GLP-1RAs is attributed to the restoration of mitochondrial function, reduction of oxidative stress, and prevention of subsequent apoptosis. Ex-4 enhances the activities of key antioxidant enzymes in hippocampal mitochondria, including catalase, manganese superoxide dismutase, and glutathione peroxidase, reversing AGE-dependent decline. Ex-4 treatment upregulates the expression of the proteins critical for mitochondrial biogenesis, including peroxisome proliferator-activated receptor-γ coactivator-1α (PGC-1α), nuclear respiratory factor-1 (NRF-1), and transcription factor a, mitochondrial (Tfam). It also reduces GSK-3β activity [47]. In addition, Ex-4 provides neuroprotection against ischemia-induced damage in the hippocampus by downregulating hypoxia-inducible factor-1α and modulating apoptosis through the increased B-cell lymphoma 2 (Bcl-2)/Bcl-2-associated X-protein (Bax) ratio, which is mediated by the GLP-1 receptor [48]. The protective effects of liraglutide against Aβ-induced neurotoxicity in neuroblastoma cells are attributed to the activation of the phosphoinositide 3-kinase (PI3K)/Akt signaling pathway, as evidenced by the increased phosphorylation of Akt and the altered expression of the apoptosis-related proteins Bcl-2/Bax, and cytochrome c [49].

### Neuroinflammation

Neuroinflammation is a critical pathogenesis of neurodegeneration, and diabetes significantly and systemically enhances inflammation, including that in the brain. EX-4 affects the microglia (immune cells in the brain) through an autocrine mechanism, in which interleukin-10 (IL-10) mediates β-endorphin expression. EX-4 stimulates the expression of anti-inflammatory M2 microglial markers (including IL-10, IL-4, arginase 1, and CD206), but not that of proinflammatory M1 markers, which are linked to neuroprotection and antinociception in neuropathic rats [50]. Ex-4 significantly reduces neuroinflammation by converting proinflammatory M1 microglia into anti-inflammatory M2 microglia both in vitro and in vivo through the rescue of the expression of Arf and Rho GAP adapter protein 3 (ARAP3); this finding suggests that the PI3K/ARAP3/(Ras homolog family member A (RhoA) signaling pathway plays a crucial role in Ex-4-mediated anti-inflammation [51]. In an AD mouse model, liraglutide reduces the activation of the NLR family pyrin domain containing 3 (NLRP3) inflammasome in the microglia, leading to the decreased production of proinflammatory cytokines and Aβ plaques [52]. Semaglutide, a GLP-1RA, mitigates seizure severity and cognitive impairment through the inhibition of the lipopolysaccharide (LPS)- and nigericin-induced NLRP3 inflammasome pathway, reducing lactate dehydrogenase (LDH) release by microglial cells [53].

### Autophagy

The impairment of autophagy and the clearance of accumulated, unwanted proteins play crucial roles in the development of neurodegeneration. Liraglutide treatment results in the increased phosphorylation of AMP-activated protein kinase (AMPK) and the reduced phosphorylation of mammalian target of rapamycin (mTOR); this result suggests that autophagy pathways are activated in high-glucose-treated hippocampal neurons. Liraglutide-induced increases in the microtubule-associated protein 1A/1B-light chain 3 (LC3)-II expression and p62 degradation were prevented by the compound C-mediated inhibition of AMPK; thus, liraglutide-induced autophagy in hippocampal neurons is induced by the AMPK/mTOR pathway [54]. By enhancing the autophagy flux and reducing oxidative stress and mitochondrial dysfunction, semaglutide and liraglutide protect neuroblastoma against 6-hydroxydopamine (6-OHDA)-induced toxicity, a common toxin model of PD. Semaglutide significantly increases the levels of the autophagy-related proteins LC3-II/LC3-I, Beclin1, and autophagy-related 7 (Atg7) and decreases p62 levels, indicating enhanced autophagy [55]. Some studies have also demonstrated the neuroprotective effect of GLP-1RAs through broader mechanisms.

### Growth and apoptosis

Ex-4 significantly improves the survival of rat cortical neurons under oxygen/glucose deprivation (OGD) conditions through reduced apoptosis rates, increased expression of glucose-regulated protein 78, and decreased levels of C/EBP-homologous protein, indicating the role of Ex-4 in modulating unfolded protein response for protecting neurons from ischemic damage [56]. Ex-4 also promotes neurite outgrowth and survival in neurons in the dorsal root ganglion of adult rats through the activation of the PI3K signaling pathway, which in turn suppresses RhoA activity, a negative regulator of nerve regeneration [57]. Liraglutide protects neuroblastoma cells against neurotoxicity induced by the organophosphate pesticide mipafox by enhancing neuritogenesis, increasing glucose uptake, and elevating the levels of cytoskeleton proteins and synaptic-plasticity modulators. It also reduces the levels of the proinflammatory cytokine IL-1β and decreases caspase-3 activity [58].

**Fig. 2:**
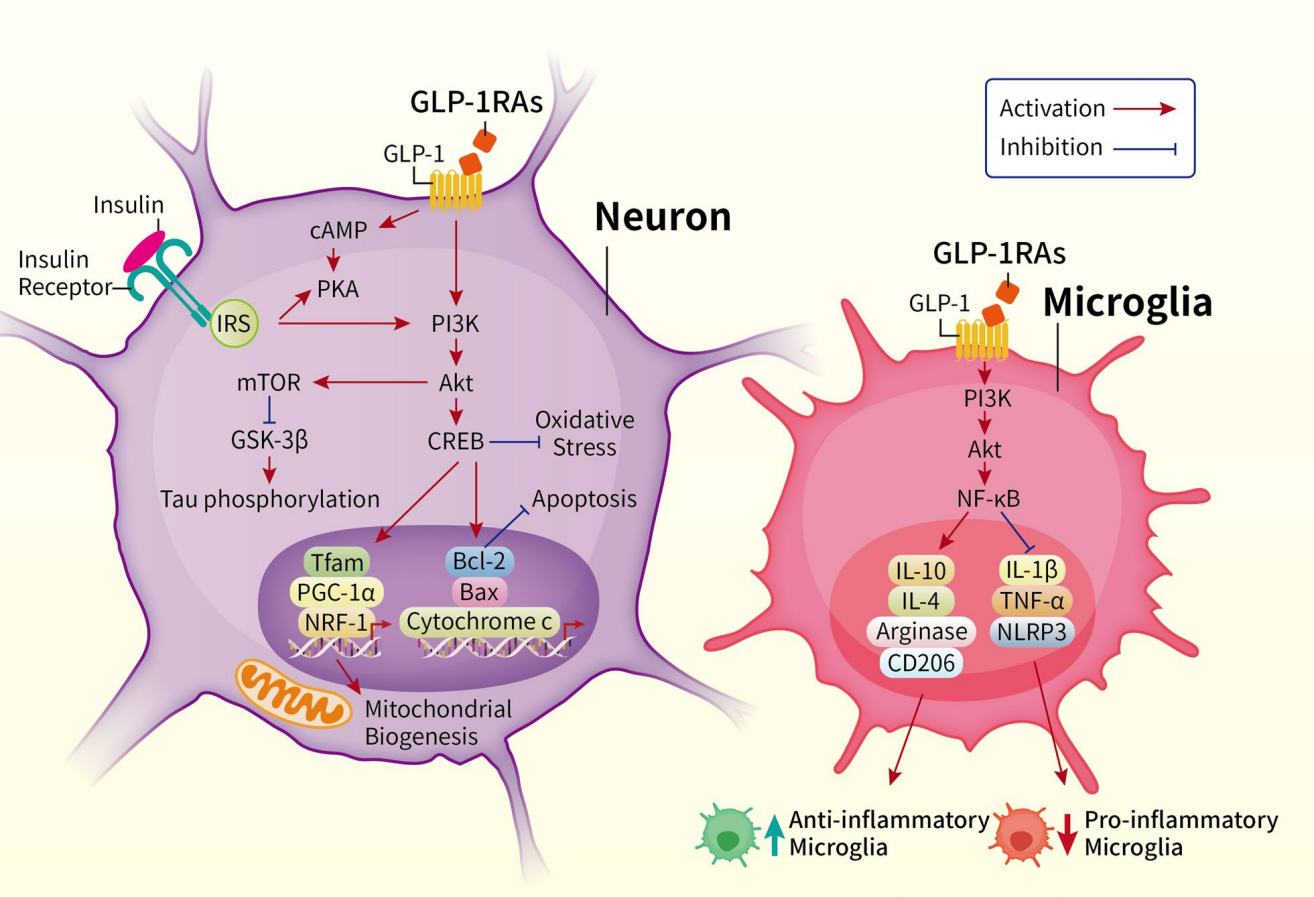
This figure illustrates the neuroprotective mechanisms of GLP-1 receptor agonists (GLP-1RAs) in neurons and microglia, highlighting the key molecular pathways involved in neurodegeneration and inflammation. In neurons, the binding of GLP-1 to its receptor (GLP-1R) activates the cyclic AMP (cAMP) and protein kinase A (PKA) signaling pathway, which in turn activates PI3K/Akt signaling. This activation leads to the downstream phosphorylation and inhibition of GSK-3β, reducing tau phosphorylation—a critical event in neurodegenerative diseases such as Alzheimer’s disease. Additionally, Akt activation promotes mitochondrial biogenesis by upregulating transcription factors such as Tfam, PGC-1α, and NRF-1, leading to enhanced energy production and reduced oxidative stress. The Akt/CREB pathway also activates Bcl-2 family proteins, inhibiting apoptosis by preventing cytochrome c release. Overall, these signaling events protect neurons from oxidative stress and apoptosis, which are central to neurodegeneration. In microglia, GLP-1R activation via PI3K/Akt signaling also reduces neuroinflammation by modulating NF-κB activity. This pathway decreases the production of pro-inflammatory cytokines, including IL-1β, TNF-α, and NLRP3, and increases anti-inflammatory markers such as IL-10, IL-4, arginase, and CD206. This shift in cytokine balance promotes the transformation of microglia into an anti-inflammatory state, which is neuroprotective, reducing overall inflammation and damage in the brain. The figure highlights the dual role of GLP-1RAs in reducing neurodegeneration by improving neuronal survival and modulating microglial activity, providing a potential therapeutic approach for neurodegenerative diseases such as AD and PD. *AGE* advanced glycation end products, *Akt* protein kinase B, *Bax* Bcl-2 associated X protein, *Bcl-2* B-cell lymphoma 2, *cAMP* cyclic adenosine monophosphate, *CREB* cAMP response element-binding protein, *GSK-3β* glycogen synthase kinase-3 beta, *IL-1β* interleukin 1 beta, *IL-4* interleukin 4, *IL-10* interleukin 10, *IRS* insulin receptor substrate, *IR* insulin resistance, *mTOR* mammalian target of rapamycin, *NF-κB* nuclear factor kappa-light-chain-enhancer of activated B cells, *NLRP3* NOD-, LRR-, and pyrin domain-containing protein 3, *NRF-1* nuclear respiratory factor 1, *PGC-1α* peroxisome proliferator-activated receptor gamma coactivator 1-alpha, *PI3K* phosphatidylinositol 3-kinase, *PKA* protein kinase A, *Tfam* mitochondrial transcription factor A, *TNF-α* tumor necrosis factor alpha

In summary, in in vitro models, the protective mechanisms of GLP-1RAs are complex. In neurons, it involves the activation of PI3K/Akt signaling pathway, inhibition of GSK-3β, reducing tau phosphorylation, promotion of mitochondrial biogenesis, activation of the Bcl-2 family proteins, and inhibiting apoptosis. In microglia, GLP-1R activation via PI3K/Akt signaling also reduces neuroinflammation by modulating NF-κB activity, decreases the production of pro-inflammatory cytokines, increases anti-inflammatory markers such as IL-10, IL-4, Arginase, and CD206, and the transformation of microglia into an anti-inflammatory state. Thus, the dual role of GLP-1RAs in reducing neurodegeneration by improving neuronal survival and modulating microglial activity, providing a potential therapeutic approach for neurodegenerative diseases (summarized in Fig. 2).

## Neuroprotective effects of GLP-1RAs in AD, PD, and other in vivo disease models

### Effects on wild type and diabetes in vivo models

The neuroprotection of GLP-1RAs in in vivo models is underpinned by the ability of GLP-1RAs to cross the blood–brain barrier (BBB) and by the presence of GLP-1 receptors in the brain. The BBB-crossing ability of liraglutide and lixisenatide has been successfully demonstrated, and they subsequently activate GLP-1 receptors in the brain. This activation leads to neuroprotection and the promotion of neuronal progenitor proliferation [59]. Initially, the neuroprotection of GLP-1RAs was tested in in vivo models of diabetes. The subcutaneous administration of liraglutide not only improves hyperglycemia and peripheral IR but also reverses insulin signaling impairment in the brain, leading to decreased AD-associated tau hyperphosphorylation [60]. In addition, liraglutide improves learning and memory in diabetic mice [61]. Interestingly, the benefit of liraglutide for tau phosphorylation in diabetic mice cannot be replicated by insulin treatment, suggesting that the unique neuroprotective effects of GLP-1RAs are medicated by pathways other than insulin signaling pathways [62].

The chronic subcutaneous administration of Ex-4 attenuates the peripheral symptoms of type 2 diabetes in rats, including hyperglycemia and IR, and enhances the levels of brain cortical GLP-1 and insulin-like growth factor (IGF)-1 and their downstream signaling pathways. It also upregulates brain cortical autophagy and ameliorates apoptosis [63]. Ex-4 also promotes brain-derived insulin production and enhances insulin signaling through the Wnt/β-catenin/NeuroD1 signaling pathway, which subsequently reduces tau hyperphosphorylation and cognitive dysfunction in diabetic mice [64]. EX-4 treatment reduces tau hyperphosphorylation levels in the hippocampus, a key area for memory and AD development [65]. Lastly, Ex-4 also protects the AGE-induced activation of receptor AGE signaling pathway, which is responsible for the inflammation and oxidative stress induced by AGEs [66].

### Aging models

Given the close association between diabetes, aging, and neurodegeneration, GLP-1RAs have been tested in in vivo aging models. Liraglutide, but not metformin, prevents insulin-induced neuronal senescence, including abnormal electrophysiological activity, and impairs glycolysis, favoring the production of p25; p25 induces the hyperactivation of cyclin-dependent kinase 5 and disrupts GSK3β-mediated processes [67]. Ex-4 also reverses endothelial cell aging in the mouse brain, which is characterized by zonation-dependent transcriptomic changes; thus, Ex-4 treatment improves BBB integrity and reduces the microglial priming associated with aging [68]. Systemic exenatide treatment can reverse aging-associated transcriptomic changes in various brain cell types in mice, including glial and neurovascular cells. This genome-wide reversal influences the common and cell type-specific pathways involved in aging and neurodegeneration. Additionally, aging leads to a reduction in AD-associated transcriptomic signatures in the microglia [69].

### Neurodegeneration and AD models: exenatide

Considering the successful neuroprotection of GLP-1RAs in in vivo diabetic models, researchers have directly tested GLP-1RAs in in vivo models of neurodegenerative diseases, including toxin-induced and transgenic models. Ex-4 regulates intracellular calcium homeostasis and prevents neuronal loss by preventing the Aβ1-42-induced calcium overload. Ex-4 can significantly rescue memory deficits and neuropathological changes in APP/PS1 AD mice, and it can downregulate the activities of *N*-acetylglucosaminyltransferase III and bisecting *N*-acetyl-d-glucosamine through the Akt/GSK-3β/β-catenin signaling pathway [70]. Ex-4 modulates apoptotic pathways in the hippocampus through the regulation of the expression levels of Bcl2, Bax, and caspase-3 [71], and it improves mitochondrial function and integrity in AD mice through the PI3K/Akt signaling pathway [72].

Ex-4 also modulates the influx of calcium through L-type voltage-dependent calcium channels (L-VDCCs) and *N*-methyl-d-aspartate receptors, and it maintains the expression of phosphorylated calcium/calmodulin-dependent protein kinase type IIα [73]. In a mouse model of AD, combining insulin with Ex-4 leads to the significant downregulation of the expression of insulin receptor signaling pathway genes in AD mice, which is associated with improved spatial learning. However, the reduction of Aβ levels is nonsignificant [74]. In 5xFAD transgenic mice, Ex-4 treatment significantly improves cognitive impairment, reduces Aβ1-42 deposition in the hippocampus, and alleviates synaptic degradation. Additionally, it restores mitochondrial morphology, mitochondrial energy production, and mitochondrial dynamics and inhibits oxidative stress [75]. Ex-4 also reverses the high-fat-diet-induced impairment of brain-derived neurotrophic factor (BDNF) signaling and the inflammatory response in the 3xTg-AD mouse model [76]. Lastly, EX-4 enhances cognitive function in APPxPS1-Ki AD mice by improving short- and long-term memory and increasing brain lactate levels, indicating a shift toward anaerobic glycolysis. However, exenatide has no effect on 3xTg-AD mice, suggesting that its benefits are model-specific [77].

### Neurodegeneration and AD models: other GLP-1 RAs

Liraglutide effectively modulates tau hyperphosphorylation through the GSK-3β pathway; therefore, liraglutide treatment attenuates the learning and memory impairments induced by the exogenous application of Aβ1-42 in mice [78]. Long-term treatment with liraglutide significantly reduces tau pathology and improves motor function in a transgenic mouse model of tauopathy. Specifically, liraglutide treatment leads to a significant reduction in the phosphorylation of tau proteins, improving survival rates and reducing motor impairment in these mice [79]. More than neuron, liraglutide alleviates mitochondrial dysfunction in astrocytes through the cAMP/PKA signaling pathway, and it enhances the neuronal supportive capacity of astrocytes treated with Aβ [80]. However, similar to Ex-4, the responsiveness of liraglutide differs between AD models; further research is warranted to understand the mechanisms underlying these differences [81]. Lastly, in a nonhuman primate model of AD, liraglutide provides partial protection, reducing AD-related insulin receptor, synaptic, and tau pathology in specific brain regions [82].

Other novel GLP-1RAs have also been tested, whether used singly or in combination with other drugs. Treatment with liraglutide and a lipidized analog of prolactin-releasing peptide (Palm11-PrRP31) exerts neuroprotective effects in APP/PS1 mice. This combination treatment significantly reduces the Aβ plaque load, neuroinflammation, and tau phosphorylation in the brain [83]. In APP/PS1 transgenic mice, (D-Ser2)Oxm, a dual GLP-1 and Gcg receptor agonist, notably improves spatial memory and synaptic plasticity, reduces Aβ plaque deposition in the hippocampus, and modulates the PI3K/AKT1/GSK3β signaling pathway [84]. NLY01, an engineered Ex-4, effectively blocks the Aβ-induced activation of the microglia and prevents the conversion of the microglia into reactive astrocytes in transgenic AD mouse models (5xFAD and 3xTg-AD) [85]. A novel GLP-1/GIP/Gcg triagonist significantly improves cognitive deficits and reduces AD pathology in 3xTg-AD mice. This treatment also enhances long-term spatial memory, working memory, and hippocampal synaptic plasticity. It also decreases Aβ and phosphorylated tau aggregates in the hippocampus [86].

### Neurodegeneration and PD models

The neuroprotection of GLP-1RAs has also been tested in in vivo models of PD, the second most common neurodegenerative disease. Mice with PD induced by 1,2,3,6 tetrahydro-1-methyl-4-phenylpyridine (MPTP) were treated with liraglutide and lixisenatide, which are GLP-1RA mimetics. Both drugs prevented MPTP-induced motor impairment, protected dopaminergic neurons in the substantia nigra, reduced the expression of the proapoptotic signaling molecule Bax, and increased the levels of the antiapoptotic molecule Bcl-2 [87]. In MPTP-induced PD models, liraglutide treatment reduced the levels of neuroinflammatory markers in the substantia nigra through AMPK activation and NF-κB reduction [88]. In toxin-induced PD mouse models, DA-JC1, a novel dual GLP-1/GIP receptor agonist, improved motor impairment, increased the number of dopaminergic neurons in the substantia nigra, enhanced BDNF levels, and modulated apoptosis signaling pathways by altering the expression of Bax and Bcl-2 [89, 90].

In summary, the neuroprotective effects of GLP-1RAs have been demonstrated in various in vivo models of diabetes, aging, AD, and PD. GLP-1RAs can cross the blood–brain barrier and activate brain GLP-1 receptors, leading to neuroprotection by improving insulin signaling, reducing tau hyperphosphorylation, and enhancing cognitive function in diabetic and aging models. Exenatide (Ex-4) and liraglutide are effective in modulating key pathways involved in neurodegeneration, such as apoptosis, mitochondrial function, and autophagy, in models of AD and PD. While the results in AD models are mixed, with benefits varying between specific models, GLP-1RAs show consistent neuroprotective effects in PD models, protecting dopaminergic neurons and reducing inflammation.

## Clinical studies on neuroprotection of GLP-1RAs

After numerous preclinical studies have examined the neuroprotective effects of GLP-1RAs, new clinical trials have been initiated. These trials have targeted various groups, including individuals with diabetes, focusing on cognitive effects, and those with or at risk of AD or PD (summarized in Table 2 for AD and Table 3 for PD).

**Table 2:** Summary of registered clinical trials of glucagon-like peptide-1 receptor agonists (GLP-1 RAs) on cognition, mild cognitive impairment (MCI) and Alzheimer’s disease (AD)

GLP-1 RAs	Population	Treatment duration	Phase	Outcome measurements (primary**/**secondary)			Status	ClinicalTrials.gov identifier	Ref.
				Clinical	Imaging	Biomarkers			
Exenatide	MCI or early AD	18 months	II	Tolerability/MMSE, ADAS-Cog, CDR	Nil	CSF (tau, p181 tau, Aβ42)	Terminated	NCT01255163	[95]
Exenatide	MCI and dysglycemia/prediabetes	32 weeks	III	ADAS-Cog/MMSE, VF, GDS, CDR, NPI, ADL, IADL	Nil	Nil	Unknown	NCT02847403	Nil
Liraglutide	MMSE > 27	12 weeks	N/A	BVRT, CVLT-II, D-KEFS, RCFT, PP, WAIS-III, WASI	Resting state fMRI	Nil	Completed	N/A	[94]
Liraglutide	Aging adults with prediabetes	90 days	I	ACT, BVRT, BNT, BFSRT, D-KEFS, color-word subtest, PP, ROCFT, TCFT, WASI, WAIS	Nil	Nil	Completed	NCT02140983	Nil
Liraglutide	Bipolar disorders or depressive disorders with below-average performance in the TMT-B	4 weeks	III	TMT-B	Resting state fMRI	Nil	Completed	NCT02423824	Nil
Liraglutide	HIV and TIIDM	6 months	IV	Global cognitive scores	Nil	Nil	Terminated	NCT02743598	Nil
Liraglutide	TIIDM	12 weeks	III	DST, RAVL, LDFR, TMT, ANT, CDT, MMSE, MES	Functional NIRS	Nil	Completed	NCT03707171	[92]
Liraglutide	TIIDM, MMSE > 24	16 weeks	N/A	MoCA	Change of olfactory brain activation by fMRI	Nil	Completed	NCT03961659	[91]
Liraglutide	TIIDM with MCI	48 weeks	N/A	MoCA/RBANS	fMRI	Nil	Recruiting	NCT05313529	Nil
Liraglutide	TIIDM	12 weeks	III	MMSE	Nil	Blood (tau, p181 tau, Aβ42, TDP-43, IL-6, IL-8, TNF-α)	Completed	NCT05360147	Nil
Dulaglutide	TIIDM, HbA1c ≤ 9.5%, age > 50 years old	8 years	III	MoCA, DSST	Nil	Nil	Completed	NCT01394952	[93]
Dulaglutide	Bipolar disorders	24 weeks	N/A	CBCT	Nil	Nil	Recruiting	NCT06331286	Nil
Semaglutide	Major depression disorder	16 weeks	II	DSST, stroop test, spatial n-back/RAVLT, TMT-A, PDQ	Nil	Nil	Recruiting	NCT04466345	Nil
Semaglutide	MCI and metabolic syndrome	12 months	II	Trails B, DST, CF, ADAS-Cog, LMS I-WMT/CDR, ADL, IADL	CBF, FDG-PET/WHH and GM in MRI	Plasma Aβ42/Aβ40 ratio, p-tau181, p-tau 231, NfL, GFAP	Recruiting	NCT06072963	Nil
Semaglutide	Healthy volunteers	Single dose	N/A	**Reward**/emotional processing, emotional impulsivity, memory	Nil	Nil	Recruitment pending	NCT06363487	Nil

*ADAS-Cog* Alzheimer’s disease assessment scale-cognitive subscale, *ADL* activities of daily living, *ANT* attention network test, *BDNF* brain-derived neurotrophic factor, *BFSRT* Buschke–Fuld selective reminding test, *BNT* Boston naming test, *BVRT* Benton visual retention test, *CBCT* Chinese brief cognitive test, *CBF* cerebral blood flow, *CDR* clinical dementia rating, *CDT* clock-drawing test, *CF* category fluency, *CSF* cerebrospinal fluid, *CT* computed tomography, *CVLT-II* California verbal learning test second edition, *D-KEFS* Delis–Kaplan executive function system, *DSST* digit symbol substitution test, *DST* digital symbol test, *FDG-PET* fluorodeoxyglucose-positron emission tomography, *fMRI* functional magnetic resonance imaging, *GDS* geriatric depression scale, *GFAP* Glial fibrillary acidic protein, *GM* grey matter, *IADL* instrumental activities of daily living, *IL-6* interleukin 6, *IL-8* interleukin 8, *LDFR* long delay free recall, *LMS I-WMT* logical memory story I from the Wechsler memory test, *M1* primary motor cortex, *MES* memory and executive screening, *MMSE* mini-mental status examination, *MoCA* Montreal cognitive assessment, *NfL* neurofilament light chain, *NIRS* near-infrared spectroscopy, *NPI* neuropsychiatric inventory, *PHQ-9* patient health questionnaire, *PP* Purdue pegboard, *RAVL* Rey auditory verbal learning, *RBANS* repeatable battery for the assessment of neuropsychological status, *RCFT* Rey complex figure test and recognition trial, *ROCFT* Rey–Osterrieth complex figure test, *SMA* supplementary motor area, *TCFT* Taylor complex figure task, *TDP-43* TAR DNA-binding protein 43,*TMT* trail making test, *TNF-α* tumor necrosis factor alpha, *VF* verbal fluency, *WAIS-III* Wechsler adult intelligence scale-third edition, *WASI* Wechsler abbreviated scale of intelligence, *WLM* Wechsler logical memory, *WMH* White matter hyperintensity

**Table 3:** Summary of registered clinical trials of glucagon-like peptide-1 receptor agonists (GLP-1 RAs) on Parkinson’s disease (PD)

GLP-1 RAs	Population	Treatment duration	Phase	Outcome measurements (primary**/**secondary)			Status	ClinicalTrials.gov identifier	Ref.
				Clinical	Imaging	Biomarkers			
Exenatide	PD H&Y 2 to 2.5	48 weeks	II	MDS-UPDRS III/UDRS, MADRS, PDQ39, NMSS, LED	DaTSCAN	CSF-BDNF	Completed	NCT01971242	[97]
Exenatide	Not applicable	1 year	I	Nil	Free-water accumulation in the substantia nigra, BOLD signal in the posterior putamen, M1, and SMA	Nil	Completed	NCT03456687	Nil
NLY01, a pegylated form of exenatide	PD	36 weeks	II	MDS UPDRS II and III	Nil	Nil	Active, not recruiting	NCT04154072	[98]
Exenatide	PD H&Y ≤ 2.5	2 years	III	MDS UPDRS III/MDS UPDRS I, II, VI, PET, MoCA-K, K-PDQ39, K-NMSS, LED, H&Y	Nil	Nil	Active, not recruiting	NCT04232969	Nil
Exenatide, sustained release	PD H&Y ≤ 2.5	48 weeks	II	MDS UPDRS III/MDS UPDRS I, II, VI, timed walk assessment, MoCA, UDRS, PHQ-9, PDQ39, NMSS, LED	Nil	Nil	Unknown	NCT04269642	Nil
Exenatide	PD H&Y ≤ 2	18 months	II	MDS UPDRS I–VI, H&Y, MoCA, PDQ-39, NMSQuest, LED, ESS, MADRS, B-SIT	FDG-PET network analysis	Nil	Active, not recruiting	NCT04305002	Nil
Liraglutide	PD within 2 years of diagnosis	54 weeks	II	UPDRS III, NMSS, MDRS-2/PDQ39, UPDRS I, II, VI	Nil	Nil	Completed	NCT02953665	Nil
Semaglutide	PD	48 months	II	MDS-UPDRS III in OFF	Nil	Nil	Recruitment pending	NCT03659682	Nil
Lixisenatide	PD H&Y < 3, within 3 years of diagnosis	12 months	II	MDS-UPDRS III	Nil	Nil	Completed	NCT03439943	[99]

*B-SIT* brief smell identification test, *DaTscan* dopamine transporter scan, *LED* levodopa equivalent dose, *M1* primary motor cortex, *MADRS* montgomery-asberg depression rating scale, *MDRS-2* mattis dementia rating scale 2, *MDS* movement disorder society, *MES* memory and executive screening, *MMSE* mini-mental status examination, *MoCA* montreal cognitive assessment, *NMSQuest* non-motor symptoms questionnaire, *NMSS* non-motor symptoms scale for Parkinson’s disease, *PDQ39* Parkinson’s disease questionnaire-39, *PET* positron emission tomography, *SMA* supplementary motor area, *UDRS* unified dykinesia rating scale, *UPDRS* unified Parkinson’s disease rating scale

### Cognition-orientated clinical trials: people with diabetes

In patients with type 2 diabetes without dementia [defined by a Mini Mental Status Examination (MMSE) score of > 24], treatment with liraglutide, but not dapagliflozin or acarbose, over 16 weeks significantly enhanced impaired odor-induced hippocampal activation, as assessed by functional magnetic resonance imaging (fMRI). Although no significant improvement was found in overall cognitive performance, as evaluated by the MMSE and the Montreal Cognitive Assessment (MoCA), notable enhancements were observed in the delayed memory, attention, and executive function subdomains of the MoCA. These enhancements were speculated to be a direct effect of liraglutide, rather than an effect of decreased blood glucose, which was achieved by dapagliflozin and acarbose [91]. Similarly, a prospective, parallel-assignment, open-label, phase III study examined changes in MMSE scores following 12-week liraglutide treatment in individuals with diabetes (glycated hemoglobin [HbA1c] > 7.0% and on oral antidiabetic drugs or insulin for at least 3 months). Multiple cognitive assessments were conducted, involving memory, executive function, attention, and verbal function evaluation. Compared with the control group, the liraglutide group derived greater cognitive benefit on the improvements in MMSE scores and category naming scores. Additionally, liraglutide significantly enhanced brain activation in the dorsolateral prefrontal cortex and orbitofrontal cortex during a verbal fluency task. The increases in blood flow and brain activation in the dorsolateral prefrontal cortex and orbitofrontal cortex were strongly associated with improvements in cognitive performance [92].

The Researching Cardiovascular Events with a Weekly Incretin in Diabetes (REWIND) trial focused on adults aged ≥ 50 years with either established or newly diagnosed type 2 diabetes and additional cardiovascular risk factors, and this trial investigated subjects with HbA1c ≤ 9.5%, receiving up to two oral glucose-lowering drugs with or without basal insulin, and a body mass index of at least 23 kg/m^2^. The REWIND trial mainly assessed the cardiovascular outcomes of dulaglutide over up to 8 years. Interestingly, the trial also revealed a positive correlation between dulaglutide treatment and a reduced risk of cognitive impairment. With the adjustment for baseline cognition, participants who had undergone dulaglutide treatment for approximately 5 years exhibited a 14% lower hazard ratio of substantive cognitive impairment (SCI) adjusted for baseline scores during follow-up [93].

### cognition-orientated clinical trials: people with risk or diagnosed AD

In a clinical trial in individuals at risk of AD, participants aged 45 to 70 years who did not have diabetes or a history of neurological disorders were recruited. All participants exhibited SCI but maintained a MMSE score of ≥ 27. The results revealed that 12-week treatment with liraglutide led to significant improvements in intrinsic connectivity within the default mode network, as assessed by resting-state fMRI. Despite these changes on neuroimages, no observable cognitive differences were noted between the treatment and placebo groups over the study period. These findings highlight the potential of liraglutide to enhance brain connectivity in populations at risk of AD, suggesting its neuroprotective effect [94]. Regarding the participants with MCI or AD, defined based on a clinical dementia rating (CDR) global score of 0.5 or 1, although the 18-month treatment with exenatide was safe and well-tolerated, it did not significantly affect clinical and cognitive measures, nor did it affect MRI cortical thickness and volume or most biomarkers in the cerebrospinal fluid (CSF), plasma, and plasma neuronal extracellular vesicles (EVs). The only notable positive outcome was the reduction of Aβ42 in plasma neuronal EVs, which may reflect the neuronal condition in the systemic circulation. Unfortunately, the study was prematurely terminated and underpowered, which precludes a conclusive determination of the disease-modifying effects of exenatide for MCI or early AD in clinical settings [95].

### PD clinical trials

The neuroprotective effects of GLP-1RAs, particularly exenatide, seem to be more promising in PD. Exenatide treatment for 12 months was generally well-tolerated in individuals with mild PD, which was defined as Hoen and Yahr (H&Y) stages 2 to 2.5. PD symptoms significantly improved, including motor function assessed by the Movement Disorder Society-Unified Parkinson’s Disease Rating Score (MDS-UPDRS) Part III motor scores and cognitive function assessed by the Mattis Dementia Rating Scale-2 (Mattis DRS-2). These improvements were noted in the exenatide treatment group compared with the control group of PD patients. Crucially, these improvements persisted even after a 2-month washout period, suggesting the potential long-term benefits [96]. A subsequent larger-scale phase II study further investigated the effect of exenatide on PD. Participants were treated with exenatide for 48 weeks. The study found a statistically significant improvement in off-medication MDS-UPDRS Part III motor scores; at the end of the treatment, a 2.3 point improvement was noted in the exenatide group compared with a 1.7 point deterioration in the placebo group. This improved score persisted after a 12-week washout period as well. However, no significant differences were detected in other outcomes, including nonmotor symptoms, cognition, finger tapping, walking speed, and levodopa equivalent dose [97]. By analyzing serum neuron-derived EVs, the study further explored how exenatide may modify brain insulin signaling pathways. Exenatide treatment led to the augmented activation of proteins involved in insulin signaling in the brain and of effectors, such as Akt and mTOR. This increased activation was correlated with clinical improvements in PD symptoms. These findings suggest the potential neuroprotective effects of exenatide and indicate its utility as a biomarker of the treatment response in PD [98]. However, NLY01, a brain-penetrant, pegylated, longer-lasting version of exenatide, provided no significant benefits in PD patients with a H&Y stage of ≤ 2.5 and an average disease duration of 1 year, as assessed by the MDS-UPDRS Parts II and III scores. A subgroup analysis revealed significant improvement in PD patients aged < 60 years, suggesting the age-dependent efficacy of NLY01 in PD [99].

A phase II RCT of lixisenatide in early PD, which was defined as patients recruited within 2 years of diagnosis, also demonstrated the promising disease-modifying effect of lixisenatide, particularly regarding motor disability. After treatment for 12 months, MDS-UPDRS Part III scores improved slightly by 0.04 points in the lixisenatide group and decreased by 3.04 points in the placebo group. Following a 2-month washout period, the mean MDS-UPDRS motor scores in the off-medication state were 2.9 points higher in the placebo group, underscoring the potential benefits of lixisenatide in early PD [100]. These results indicate a class effect of GLP-1RAs on neuroprotection in PD. At present, a large-scale phase III RCT of 2-year exenatide treatment in patients with PD, defined as a Hoen and Yahr (H&Y) stage of ≤ 2.5, is ongoing; the results are expected to be reported in 2025.

## Summary of completed and ongoing clinical trials of GLP-1RAs for neurodegeneration

In summary, clinical trials investigating GLP-1RAs in neurodegenerative diseases have yielded contrasting outcomes. In individuals with AD, GLP-1RAs did not exert disease-modifying effects on cognition. However, in patients with diabetes, treatment with GLP-1RAs offered cognitive benefits beyond their blood glucose-lowering effect. In PD, several GLP-1RAs have demonstrated promising disease-modifying effects in phase II RCTs, and the results of phase III trials are anticipated. These discrepancies might be attributed to the timing of GLP-1RA intervention in the disease course. AD is characterized by a decade-long prodromal stage, and dementia diagnosis typically occurs in the late biological stage of AD, by which time substantial amounts of Aβ and tau proteins have accumulated in the cortex [101]. Individuals at risk of AD or with diabetes may be in the early or middle biological stage of AD, offering a greater opportunity for GLP-1RAs to provide neuroprotection. By contrast, PD, which also has a prolonged prodromal stage similar to AD before the onset of motor symptoms, has a different progression of Lewy body pathology, which may take another decade to pervade the entire brain and cause significant, widespread neurodegeneration [102]. Moreover, the complex pathogenesis of PD makes it a suitable target for GLP-1RAs, which modulate numerous intracellular signaling pathways, as demonstrated by in vitro and in vivo studies.

## Challenges and future directions

Currently, GLP-1RAs have shown potential for mitigating neurodegeneration. Regarding the underlying mechanism, IR in the brain significantly contributes to neurodegenerative processes, and GLP-1RAs modulate downstream signaling pathways, including mitochondrial biogenesis, autophagy, inflammation, and cell survival. Additionally, the safety and tolerability profiles of GLP-1RAs have been well-established; thus, GLP-1RAs are favorable candidates for repurposing for the treatment of neurodegenerative diseases. However, several challenges remain. Unfavorable outcomes from AD clinical trials underscore the importance of the timing of treatment; GLP-1RAs may not effectively reverse disease progression in mid-to-late stages. Moreover, the persistence of GLP-1RAs’ beneficial effects remains uncertain. In PD clinical trials, the beneficial effects have been observed for only 1 year—a substantially short period considering the lifelong, progressive nature of neurodegeneration. Thus, future clinical trials must assess the long-term effect of GLP-1RA treatment. Lastly, the systemic effects of GLP-1RAs, such as body weight loss [103] and potential reduction in muscle mass [104], pose significant challenges for elderly individuals with neurodegenerative diseases. Lastly, the limited evidence on the effects of GLP-1 RAs on key pathognomonic proteins, Aβ and α-synuclein, weakens the scientific basis for considering GLP-1 RAs as major game-changers for neurodegenerative diseases. These factors necessitate the careful consideration of the use of GLP-1RAs in the broader context of managing and treating these neurodegenerative diseases.

The design of future clinical trials for GLP-1 receptor agonists (GLP-1RAs) targeting AD and PD may necessitate distinct approaches. In the context of AD, the focus should be on individuals at risk, such as those with MCI, subjective cognitive decline, elders with positive Aβ or tau biomarkers, *APOE*-ε4-carriers or healthy elderly individuals with high-risk scores [105]. Given the pronounced effect of tau elimination observed in in vitro and in vivo studies, selecting patients who test positive for tau biomarkers (CSF, blood, or positron emission tomography) could enhance the likelihood of achieving significant disease modification. Currently, AD patients with positive tau pathology exhibit poor responsiveness to anti-Aβ antibody treatments, which are ineffective in reducing tau levels [106]. In addition, it is recommended that the duration of future clinical trials of GLP-1RAs for AD extend to 2 to 3 years to evaluate long-term outcomes, including changes in cognition, activities of daily living, instrumental activities of daily living, and biomarkers for tau and Aβ.

In contrast, current promising clinical trials for PD primarily focus on early-stage patients with short disease duration and H&Y stage of ≤ 2.5. It may also be beneficial to consider individuals at high risk for prodromal PD. However, unlike the relatively predictable conversion from MCI to AD, the progression from hyposmia or rapid eye movement sleep behavior disorder to PD is variable [107], complicating the assessment of GLP-1RAs’ efficacy in preventing phenotype conversion. The development of novel techniques for ultra-sensitive α-synuclein detection, particularly real-time quaking-induced conversion [108], could improve the accuracy of identifying prodromal PD and serve as reliable biomarkers for treatment response. In addition, *LRRK2* G2019S carriers without clinical diagnosed PD may also be ideal as prodromal PD candidates for clinical trials.

In summary, future clinical trials should prioritize enrolling individuals at risk, followed by those in the early stages of the disease, as this offers a higher likelihood of demonstrating neuroprotective and disease-modifying effects. GLP-1 RAs may need to be combined with other agents targeting the clearance of Aβ or α-synuclein to achieve a more effective disease-modifying outcome. For AD specifically, given the well-established effects of GLP-1 RAs on tau hyperphosphorylation, we recommend that future GLP-1 RA clinical trials focus on the tau-positive subgroup of patients.

## Conclusion

Beyond their established role as potent antihyperglycemic agents, GLP-1RAs show potential for treating neurodegenerative diseases with disease-modifying effects. Both in vivo and in vitro studies provide substantial evidence of the neuroprotective benefits of GLP-1RAs through the modulation of inflammation, mitochondrial function, ROS, tau hyperphosphorylation, and anti-apoptotic pathways. However, while clinical trial results have been promising for PD, they have been less favorable in AD. In future RCTs, key considerations such as the optimal timing of intervention, treatment duration, specific patient subgroups based on molecular diagnosis, and potential systemic adverse events require further investigation.

## Data Availability

Not applicable due to the nature of review. No new data was generated.
